# Frontal and occipital-parietal alpha oscillations distinguish between stimulus conflict and response conflict

**DOI:** 10.3389/fnhum.2015.00433

**Published:** 2015-08-06

**Authors:** Dandan Tang, Li Hu, Yi Lei, Hong Li, Antao Chen

**Affiliations:** ^1^School of Education Science, Zunyi Normal CollegeZunyi, China; ^2^Key Laboratory of Cognition and Personality (Ministry of Education) and School of Psychology, Southwest UniversityChongqing, China; ^3^Research Centre for Brain Function and Psychological Science, Shenzhen UniversityShenzhen, China

**Keywords:** flanker task, event-related potential (ERP), neural oscillation, alpha band, practice paradigm

## Abstract

Conflicts between target and distraction can occur at the level of both stimulus and response processing. However, the neural oscillations underlying occurrence of the interference in different levels have not been understood well. Here, we reveal such a neural oscillation modulation by combining a 4:2 mapping design (two targets are mapped into one response key) with a practice paradigm (pretest, practice, and posttest) when healthy human participants were performing a novel color-word flanker task. Response time (RT) results revealed constant stimulus conflict (SC, stimulus incongruent minus congruent, SI-CO) but increased response conflict (RC, response incongruent minus stimulus incongruent, RI-SI) with practice. Event-related potential (ERP) results demonstrated stable P3 amplitude differences for the SI-CO in the centro-parietal region across practice, which may reflect maintenance of the stimulus processing; and significantly larger P3 amplitudes in the same region for the RI relative to SI trial type in posttest, which may reflect inhibition of the distraction response. Further, neural oscillatory results showed that with practice, the lower alpha band in the frontal region and the upper alpha band in the occipital-parietal region distinguished between stimulus- and response-conflicts, respectively, suggesting that practice reduces the alertness (sensitiveness) of the brain to conflict occurrence, and enhances stimulus-response associations.

## Introduction

A basic research phenomenon in cognitive control is the so-called congruency effects (simply, conflicts), which can be observed in various congruency tasks (e.g., Stroop task; Stroop, 1935, Eriksen flanker task; Eriksen and Eriksen, 1974, and Simon task; Simon, 1990). Notably, conflicts had been confirmed to occur at both stimulus processing and response output levels (Kornblum et al., 1990; Zhang et al., 1999; van Veen et al., 2001), and accordingly classified as stimulus conflict (SC) and response conflict (RC). The 4:2 mapping design (Eriksen and Eriksen, 1974; De Houwer, 2003) has been frequently used to separate the two types of conflicts (van Veen and Carter, 2002, 2005; De Houwer, 2003; Wendt et al., 2007; Chen et al., 2011, 2013a,b). For example, in a word flanker task with horizontally arranged word triads (in Chinese, and in black font) as the stimuli (Chen et al., 2013b), words “RED” and “YELLOW” were assigned to a left response, and “BLUE” and “GREEN” to a right response. Further, three trial types were established: (1) congruent (CO, all three words were identical, e.g., “RED RED RED”); (2) stimulus incongruent (SI, flanking words differed from center word but they were mapped into the same response, e.g., “RED YELLOW RED”); and (3) response incongruent (RI, flanking words differed from center word and they were mapped into different responses, e.g., “RED BLUE RED”). Based on this design, SC and RC could be measured by the response time (RT) difference of SI minus CO, and of RI minus SI, respectively.

Undoubtedly, classification is helpful for us to understand one phenomenon. Specifically, through classifying conflicts into the SC and RC, we can investigate the distinct influences of stimulus processing and response execution on the generation of conflicts (De Houwer, 2003; Chen et al., 2013b). In addition, practice is one crucial manipulation to reveal the causes of conflicts (MacLeod, 1991, 1998). Therefore, combining the practice design (pretest, practice, and posttest) and the 4:2 mapping design, Chen et al. (2013a,b) studied the effects of practice on the SC and RC. One novel and interesting finding is that, in the flanker task, the practice enlarged the size of RC but did not change that of SC (Chen et al., 2013b). In terms of the parallel distributed processing model Cohen et al. (1990) and Chen et al. (2013b) suggested that the practice may enhance the strength of processing pathway. Usually, the competition (conflict) between two strong pathways would be larger than that between two weak pathways; therefore, the RC in the posttest relative to pretest was larger. However, the study of Chen et al. (2013b) just recorded behavioral data, which cannot directly address the questions that: (1) why practice influences the two kinds of conflicts differently; and (2) how the strength of pathway processing is enhanced by practice. In the present study, we employed the same task and design as that used in the study of Chen et al. (2013b), and recorded the electroencephalography (EEG) data of participants, by which we aimed to address the above questions.

With the high temporal resolution, EEG measures can provide invaluable insights for the on-going brain activities. Apart from the well-known event-related potentials (ERPs), which are time- and phase-locked in time domain (Luck, 2005), the neural oscillatory activities that are time- and non-phase-locked in time-frequency domain are receiving increasing attention (Pfurtscheller and Lopes da Silva, 1999; Makeig et al., 2004). Such activities are characterized by either transient decreases (event-related desynchronization, ERD) or transient increases (event-related synchronization, ERS), usually confined to a specific frequency band (Pfurtscheller and Aranibar, 1977; Pfurtscheller and Lopes da Silva, 1999). ERD and ERS may denote behavioral conflicts in the congruency tasks (Compton et al., 2011, 2012; Tang et al., 2013). In the present study, combining ERP analysis with time-frequency analysis of EEG data, we aimed to examine the neural correlates of the practice-related modulations on the SC and RC.

In the flanker task, an ERP positive component (P3) peaking at 0.35–0.5 s post-stimulus presentation has been recorded in the central-parietal region and the amplitudes are larger for the incongruent compared to congruent trial type (Clayson and Larson, 2011; Frühholz et al., 2011). P3 generation may indicate a process of response inhibition (Enriquez-Geppert et al., 2010; Frühholz et al., 2011) or relate to monitoring responses to appropriate stimulus classification (Verleger et al., 2005). Moreover, P3 latencies increase when categorization of the stimulus becomes more difficult (Kok, 2001). Therefore, P3 latencies represent a measurement corresponding to stimulus evaluation time (Kutas et al., 1977). Specifically, P3 latencies are longer in the incongruent relative to congruent trial type in the flanker task (Purmann et al., 2011), which indicates that the stimulus categorization is more difficult for the incongruent relative to congruent trial type. Since P3 amplitudes and latencies index the flanker effect, one focus of the present study is to examine the effects of practice and trial type on this component.

For the neural oscillations, alpha activity (7–13 Hz) is well known as that it is associated with attentional processing and cognitive control (Klimesch, 1999; Carp and Compton, 2009; Rohenkohl and Nobre, 2011; Tang et al., 2013; Wang et al., 2015; Wu et al., 2015). Specifically, alpha activity has been suggested to modulate behavioral conflicts in the congruency tasks (Compton et al., 2011; Bonnefond and Jensen, 2012; Tang et al., 2013; Zhao et al., 2014). Moreover, alpha oscillation could be further divided into upper alpha band (11–13 Hz) and lower alpha band (7–10 Hz). The upper alpha has been reported to reflect the search and retrieval processes in semantic long-term memory (Klimesch et al., 1997; Klimesch, 1999) and endogenous control (Wu et al., 2015); the lower alpha is critically related to attentional demands such as alertness (Klimesch et al., 2007). Thus, P3 and alpha activity are two main concerns of the present study, by which we could demonstrate the neural correlates of the practice modulations on the flanker effect.

## Materials and Methods

### Participants

Thirty-one right-handed healthy volunteers (21 females), between 18 and 26 years old (21.52 ± 2.12, mean ± SD), took part in the experiment. All volunteers reported normal or corrected-to-normal vision and normal color perception. All volunteers gave written informed consent and were paid for their participation. The local ethics committee of Southwest University (Chongqing, China) approved the procedure. In addition, the volunteers were unaware of the experimental purpose.

### Stimuli and Task

The stimuli were presented in white against a black background using E-Prime software (Psychology Software Tools, Inc., Pittsburgh, PA, USA) on a 17-in computer monitor. The viewing distance was approximately 0.6 m. Responses were registered using a standard QWERTY keyboard. The stimuli consisted of words (“RED”, “GREEN”, “YELLOW”, and “BLUE”). In each trial, three horizontal-arranged words were presented in Chinese (Song Ti font) with a central target word was flanked by a distractor word on each side. The distractor words were always identical to each other. The target word was either identical to the flanking words or different from them.

The participants were instructed to respond to the central target word: (1) “RED” or “GREEN” by pressing the “Q” key with the left forefinger; and (2) “YELLOW” or “BLUE” by pressing the “P” key with the right index finger. They were instructed to perform the task as fast as possible without sacrificing accuracy. According to the congruency of the target and flanking words, three trial types were introduced, i.e., CO (e.g., “RED RED RED”), SI (e.g., “RED GREEN RED”), and RI (e.g., “RED YELLOW RED”).

### Procedure and Design

In each trial, the stimuli were presented as follows: (1) a white fixation “+” for 0.3 s; (2) a blank interval for 0.8–1 s (the interval varied randomly); (3) three horizontal-arranged words until a response was made or for 1.5 s if there was no response made; and (4) a blank interval for 0.8–1.2 s (interval varied randomly). Presentation order of the trials was randomized.

Participants performed a block of 16 trials prior to completion of seven experimental blocks. The first block and the seventh block respectively served as the pre- and post-practice block with 192 trials included for each. The remaining five blocks served as the practice blocks of 240 trials each, which enabled sufficient practice-related effects to emerge. In addition, for each experimental block, the proportion of the CO, SI, and RI trial types was equal. There was a 2-min break between blocks.

### EEG Recording and Analysis

#### EEG Recording

The EEG data were recorded using a 64-channel Brain Products system (Brain Products GmbH, Munich, Germany; pass band: 0.01–100 Hz, sampling rate: 500 Hz) using a standard EEG cap based on the extended 10–20 system. The FCz was used as the reference channel, and all channel impedances were kept below 5 kΩ. The electro-oculographic (EOG) signals were simultaneously recorded from four surface electrodes, which were placed superior to the upper eyelid and inferior to the lower eyelid and laterally 1 cm from the outer corner of the left and right orbits to monitor ocular movements and eye blinks.

#### EEG Data Preprocessing

The analysis of EEG data only focused on the data recorded in pretest and posttest. The EEG data were preprocessed using Letswave (Mouraux and Iannetti, 2008), a free signal-processing toolbox, and EEGLAB (Delorme and Makeig, 2004), an open source toolbox running under the MATLAB environment. The EEG trials were re-referenced to the bilateral mastoid electrodes. Continuous EEG data were bandpass filtered between 1 and 30 Hz. All incorrect (3.75% of all trials) and post-error (3.54% of all trials) trials were eliminated from the following analyses. EEG epochs were extracted using two time windows. For analysis in the time domain, a 1.2-s time window ranging from −0.2 s to 1 s (pre-stimulus 0.2 s and post-stimulus 1 s) was adopted and baseline corrected using the pre-stimulus time interval (−0.2 to 0 s). For analysis in the time-frequency domain, EEG epochs were segmented in a 1.8-s time window (pre-stimulus 0.8 s and post-stimulus 1 s) and baseline corrected using the pre-stimulus time interval (−0.8 to 0 s). Data were visually inspected to identify bad epochs and then to be rejected. Trials contaminated by eye-blinks and movements were corrected using an independent component analysis (ICA) algorithm (Makeig et al., 1997; Jung et al., 2001; Delorme and Makeig, 2004). In all datasets, the removed independent components (ICs) had a large EOG channel contribution and a frontal scalp distribution. As suggested by Hu et al. (2014), the baseline correction in the time domain was a necessary step for the subsequent time-frequency analysis, since it ensured that the ICA denoising and the artifact rejection were optimal.

#### Time-Domain Analysis

For each participant and each trial type (CO, SI, and RI), average waveforms of pretest and posttest were computed, time-locked to the onset of the stimulus. Single-participant average waveforms were subsequently averaged to obtain group-level average waveforms. For each trial type (CO, SI, and RI) in the pretest and posttest, P3 mean amplitudes of each participant were measured at the centro-parietal region [(CP1+CPz+P3+P1+Pz+P2)/6] between 0.4 and 0.6 s. In addition, in the pretest and posttest, the peak latencies of P3 in the centro-parietal region for each trial type (CO, SI, and RI) were measured at the single-participant mean maximum amplitudes. The chosen electrodes and time window matched the strongest P3 activity of the current data and previous research (Polich, 2007). Moreover, averaging across multiple electrodes decreased the chance of spurious findings by increasing signal-to-noise (Cohen and van Gaal, 2013). The obtained mean amplitudes and peak latencies were respectively compared using the two-way repeated-measures analysis of variance (ANOVA), with congruency (CO, SI, and RI) and practice (pretest and posttest) as within-subjects factors. In the pretest and posttest, the group-level scalp topographies in the P3 time window for the three trial types were obtained, respectively.

#### Time-Frequency-Domain Analysis

##### Calculation of time-frequency representations (TFRs)

The TFRs were obtained from single-trial EEG epochs using a continuous wavelet transform (CWT; Mouraux and Iannetti, 2008), which was able to construct a TFR of EEG signals that offered an optimal compromise for time and frequency resolution by adapting the window width as a function of estimated frequency (Hu et al., 2010). The parameters of central frequency (*ω*) and restriction (*σ*) in the CWT were 5 and 0.15, respectively. The CWT yielded, for each time course, a complex time-frequency estimate *F(t,f)* at each point *(t,f)* of the time-frequency plane, extending from −0.8 to 1 s (in steps of 0.002 s) in the time domain, and from 1 to 30 Hz (in steps of 0.58 Hz) in the frequency domain. The resulting spectrogram, *A(t,f) = |F(t,f)|*, represented the signal power as a joint function of time and frequency at each time-frequency point. The obtained TFRs contained both phase-locked and non-phase-locked modulations of EEG signal. Further, to distinguish between phase-locked and non-phase-locked EEG responses, we respectively calculated the phase-locking value (PLV; Lachaux et al., 1999) in pretest and posttest, for each trial type (CO, SI, and RI) of each participant, as follows:

$$\text{PLV}(t,f)\text{ = }\frac{1}{N}\sum_{n=1}^{N}\frac{Fn(t,f)}{\left|Fn(t,f)\right|}-\psi (f),$$

where *N* was the number of trials and *ψ(f)* was the average PLV of the pre-stimulus interval (−0.7 to −0.1 s before the onset of the stimulation) for each estimated frequency *f*.

##### Baseline correction

Single-trial TFRs were averaged to obtain averaged TFRs, which were used to identify the modulations of ongoing EEG rhythms (event-related spectral perturbation, ERSP). For each estimated frequency, ERSP magnitudes were displayed as an increase or decrease in oscillatory power relative to the pre-stimulus interval (−0.7 to −0.1 s), which was baseline corrected according to the formula: *ER(t,f)% = [A(t,f)−R(f)] R(f)*, where *A(t,f)* was the signal power at a given time *(t)* and frequency *(f)*, and *R(f)* was the signal power averaged within the pre-stimulus interval (Pfurtscheller and Lopes da Silva, 1999). The pre-stimulus time interval (−0.7 to −0.1 s) was chosen to avoid the potential adverse influence of spectral estimates biased by windowing post-stimulus activity and padding values. In pretest and posttest, grand-average TFRs were computed for the three trial types of each participant, respectively.

##### Definition of spatial region of interest (S-ROI)

We adopted a data-driven analysis protocol to define the S-ROIs, by which the practice-related effects on the SC and RC could be dissociated. Two point-by-point two-way repeated-measures ANOVAs were respectively performed to assess the effects of the experimental factors on the stimulus-induced modulations of EEG power (expressed as ER%) and to define the S-ROIs. One used the SC contrast, with congruency (CO, SI) and practice (pretest and posttest) as factors, and another used the RC contrast, with congruency (SI, RI) and practice (pretest and posttest) as factors, to distinguish between the SC and RC spatial dynamics related to practice, respectively. Each ANOVA yielded three time-frequency topography maps of *F* values for each channel, representing the main effects of both conflicts and practice and the interaction between them. To address the problem of multiple comparisons in the point-by-point statistical analysis of time-frequency topography maps (Maris and Oostenveld, 2007), the significance level (*p* value) was corrected using a false discovery rate (FDR) procedure (Benjamini and Hochberg, 1995). Significant S-ROIs were defined based on the criteria that they had to be composed of: (1) at least two nearby significant channels (Hu et al., 2013) where the interaction between conflict and practice for either ANOVA was significant; and (2) more than 200 consecutive significant time points (0.4 s) and two consecutive frequencies. Since we were specifically interested in the interactions between conflict and practice for either ANOVA, which distinguished between the SC and RC spatial dynamics related to practice, these S-ROIs with *F*_(1,30)_ > 5 (*p* < 0.050) for the interactions were selected for the subsequent quantitative analysis in the time-frequency domain.

##### Definition of time-frequency ROI (TF-ROI) within each S-ROI

A data-driven exploratory analysis strategy was adopted to define the TF-ROIs. This was performed in the following steps and similar to the previous studies (Zhang et al., 2012; Tang et al., 2013):

1. Each point* (t,f)* of the ER% time-frequency maps was compared respectively using two point-by-point two-way repeated-measures ANOVAs to assess the effects of the experimental factors on the stimulus-induced modulations of EEG power (ER%) and to define the significant TF-ROIs within the time-frequency spectrograms of each S-ROI. One used the SC contrast and practice as factors and another used the RC contrast and practice as factors to distinguish between the SC and RC modulations of neural oscillation related to practice. Each ANOVA yielded three time-frequency maps of *F* values, representing the main effects of conflicts and practice and the interaction between the two factors, respectively. Since we were specifically interested in the interactions between conflicts and practice for either ANOVA, which distinguished between the SC and RC modulations of neural oscillations related to practice, these TF-ROIs with *F*_(1,30)_ > 5 (*p* < 0.050) for the interaction were selected for the subsequent quantitative analysis.
2. To address the problem of multiple comparisons in the point-by-point statistical analysis of TFRs (Maris and Oostenveld, 2007), the significance level (*p* value) was corrected using the FDR procedure (Benjamini and Hochberg, 1995). In addition, to control for false-positive observations (Maris and Oostenveld, 2007), significant TF-ROIs were defined based on the following criteria: (1) the detected interactions between conflicts and practice were significant at the level of *p* < 0.050 for either ANOVA; and (2) the time-frequency pixels had to cover more than two full cycles of an oscillation. Thereby, within the entire time-frequency plane obtained in each S-ROI, the TF-ROIs were defined characterizing the interactions between conflicts and practice.

Lastly, consistently with the behavioral analysis, the two-way repeated-measures ANOVA was firstly used to compare the ERSP magnitude, with congruency and practice as factors, within each defined TF-ROI and to estimate the changes in neural oscillations of each trial type with practice. To clearly estimate the changes in neural oscillations of the SC and RC with practice, the following two-way repeated-measures ANOVA was used to compare the ERSP magnitude, with SC and RC contrasts and practice as factors, within each defined TF-ROI.

## Results

### Behavioral Performances

To examine the practice modulations on the SC and RC, the RT and error rates in pretest and posttest were analyzed. All incorrect (3.75% of all trials) and post-error (3.54% of all trials) trials were discarded. From the remaining trials, RT outliers (±2.5 SDs) were removed (2.57% of all trials). Across participants, mean RT and error rates for the three trial types in pretest and posttest were summarized in Table 1.

**Table 1:** **Descriptive statistics of behavioral and EEG findings**.

	Parameters	Congruency		
	(Mean ± SD)	CO	SI	RI
Pretest	RT (ms)	610 ± 70.36	626 ± 70.21	644 ± 67.20
	error rates (%)	2.32 ± 2.50	2.97 ± 2.46	6.39 ± 5.24
	P3 amplitudes (μV)	2.30 ± 1.45	1.78 ± 1.36	2.01 ± 1.79
	P3 latencies (ms)	390 ± 30.31	385 ± 27.94	413 ± 49.96
	OP alpha (ER%)	−1.12 ± 17.98	−4.01 ± 17.73	−2.29 ± 17.07
	RF alpha (ER%)	17.88 ± 22.99	22.2 ± 25.84	19.33 ± 24.25
Posttest	RT (ms)	551 ± 64.22	562 ± 68.86	590 ± 66.67
	error rates (%)	2.65 ± 2.84	2.58 ± 2.80	5.61 ± 4.95
	P3 amplitudes (μV)	2.30 ± 1.46	2.12 ± 1.39	2.55 ± 1.44
	P3 latencies (ms)	398 ± 32.85	411 ± 40.55	429 ± 44.57
	OP alpha (ER%)	4.66 ± 14.81	2.55 ± 16.98	−2.48 ± 17.59
	RF alpha (ER%)	11.48 ± 21.25	8.41 ± 21.33	8.21 ± 24.18

*Note: (1) “CO”, “SI”, and “RI” are the congruent, stimulus incongruent, and response incongruent trial types, respectively; (2) “RT” and “SD” are response time and standard deviation, respectively; and (3) “OP” and “RF” are occipital-parietal and right-frontal regions, respectively. N = 31*.

For the RT, we first conducted a two-way repeated-measures ANOVA with congruency and practice as factors. The results revealed significant main effects of congruency, *F*_(2,60)_ = 115.56, *p* < 0.001, η^2^ = 0.794, and of practice, *F*_(1,30)_ = 34.76, *p* < 0.001, η^2^ = 0.537. However, the interaction between the two factors was not significant, *F*_(2,60)_ = 1.81, *p* = 0.173, η^2^ = 0.057. Considering the significant main effects of congruency and practice, we then run two two-way repeated-measures ANOVAs to reveal the practice modulations on the SC and RC, respectively. For the SC, CO-SI contrast (CO, SI) and practice (pretest and posttest) were used as factors. For the RC, SI-RI contrast (SI, RI) and practice (pretest and posttest) were used as factors. The results were illustrated in Table 2. These results suggested that, with practice, the RC was significantly enhanced, *t*_(30)_ = 2.26, *p* = 0.021; however, the RC kept constant, *t*_(30)_ = 1.11, *p* = 0.277 (paired-samples *t* test, two-tailed).

**Table 2:** **Test statistics for the RT**.

The main effect of congruency (CO, SI)			The main effect of practice (pretest and posttest)		
*F*_(1,30)_	*p*	η^2^	*F*_(1,30)_	*p*	η^2^
50.37	***	0.627	37.44	***	0.555
The interaction between the two factors		
*F*_(1,30)_	*p*	η^2^
1.23	0.277	0.039
The main effect of congruency (SI, RI)			The main effect of practice (pretest and posttest)		
*F*_(1,30)_	*p*	η^2^	*F*_(1,30)_	*p*	η^2^
70.64	***	0.702	34.10	***	0.532
The interaction between the two factors			*Post hoc* test		
*F*_(1,30)_	*p*	η^2^	pretest and posttest: RI > SI ***;		
5.11	0.031	0.146	SI and RI: pretest > posttest ***;		

Note: ***p < 0.001, N = 31. “CO”, “SI”, and “RI” are the congruent, stimulus incongruent, and response incongruent trial types, respectively.

For the error rates, a two-way repeated-measures ANOVA with congruency and practice as factors indicated that the main effect of congruency was significant, *F*_(2,60)_ = 23.60, *p* < 0.001, η^2^ = 0.440, but neither the main effect of practice, *F*_(1,30)_ = 0.27, *p* = 0.608, η^2^ = 0.009, nor the interaction between the two factors, *F*_(2,60)_ = 0.659, *p* = 0.521, η^2^ = 0.022, was significant. The results showed that the practice had little effect on error rates.

### EEG Effects

#### Time Domain: P3

**Figure 1:**
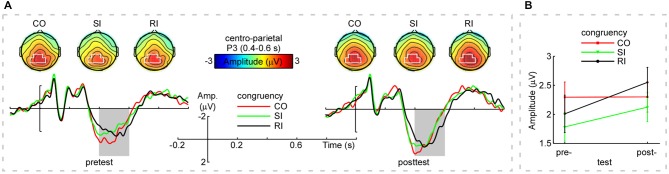
**Group-level average event-related potential (ERPs), scalp topographies, and mean amplitudes of P3**. Panel **(A; Top)**: The scalp topographies of P3 (averaged within 0.4–0.6 s) for the CO, SI, and RI trial types in the pretest and posttest (top left and top right panels, respectively). Noteworthy was that the scalp topographies of P3 displayed clear centro-parietal distribution (marked in white) for the CO, SI, and RI trial types across practice. Panel **(A; Bottom)**: The grand-average event-related potential (ERP) waveforms measured at the centro-parietal region [(CP1+CPz+P3+P1+Pz+P2)/6] for the CO, SI, and RI trial types in the pretest and posttest. *X*-axis, time (s); *Y*-axis, amplitude (μV). The vertical bars indicate the onsets of the stimuli. Note that P3 mean amplitudes were modulated by both congruency and practice in the time window of 0.4–0.6 s (outlined in gray rectangles). Panel **(B)** shows the mean amplitudes of P3 (measured at the centro-parietal region within 0.4–0.6 s for the CO, SI, and RI trial types in the pretest and posttest). Notably, practice resulted in a decreased P3 amplitude difference between SI and CO trial types, while, the difference between RI and SI trial types in P3 amplitude was increased. Error bars indicate ±1 standard errors of the mean (SEMs). Note: “Amp” is amplitude. “CO”, “SI”, and “RI” are the congruent, stimulus incongruent, and response incongruent trial types, respectively.

Figure 1A displayed the grand-average ERP waveforms (bottom) measured at the centro-parietal region [marked in white shapes, (CP1+CPz+P3+P1+Pz+P2)/6] and scalp topographies (top) measured from 0.4 s to 0.6 s for the CO, SI, and RI trial types in pretest and posttest. As observed in Figure 1A, the three trial types elicited dominant P3 at the time window of 0.4–0.6 s distributing in the centro-parietal region. We measured P3 mean amplitudes at the centro-parietal region across participants, Figure 1B and Table 1 displayed the results. For the P3 mean amplitudes, we carried out a two-way repeated-measures ANOVA with congruency (CO, SI, and RI) and practice (pretest and posttest) as factors. The results were illustrated in Table 3. Importantly, the interaction between the two factors was significant, which was embodied in significantly larger amplitudes for the CO relative to both SI and RI in the pretest, and significantly larger amplitudes for the RI relative to SI in the posttest. P3 peak latencies across participants were also summarized in Table 1. The statistical results were illustrated in Table 3. Although the main effects of congruency and practice were significant, their interaction was not.

**Table 3:** **Statistical results for the P3 mean amplitudes and peak latencies**.

	Mean amplitudes			Peak latencies		
The main effect of congruency (CO, SI, and RI)
	*F*_(2,60)_	*p*	η^2^	*F*_(2,60)_	*p*	η^2^
	5.19	0.008	0.148	14.59	***	0.327
The main effect of practice (pretest and posttest)
	*F*_(1,30)_	*p*	η^2^	*F*_(1,30)_	*p*	η^2^
	1.28	0.266	0.041	10.28	0.003	0.255
The interaction between the two factors
	*F*_(2,60)_	*p*	η^2^	*F*_(2,60)_	*p*	η^2^
	3.46	0.038	0.103	2.74	0.073	0.084
*Post hoc* tests
	pretest: CO > SI ***;		
	posttest: RI > SI, *p* = 0.010;		

Note: ***p < 0.001, N = 31. “CO”, “SI”, and “RI” are the congruent, stimulus incongruent, and response incongruent trial types, respectively.

Of note, the P3 time window (0.4–0.6 s) partly overlapped with participants’ mean RT (0.55–0.65 s). Probably, the participants’ button presses contributed to the interaction between congruency and practice. To examine this possibility, we ran the Pearson correlation analysis (two-tailed) between P3 peak latencies and RT for each trial type in the pretest and posttest. However, there was no any significant correlation (*p* > 0.11). Therefore, the significant interaction found for the P3 amplitude was not mixed with the response execution.

#### Time-Frequency Domain: Alpha-Band

**Figure 2:**
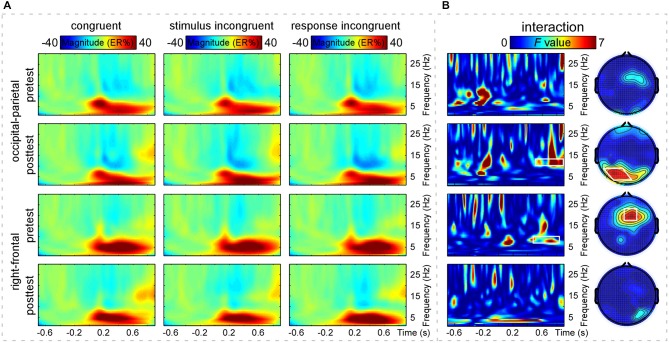
**Time-frequency distributions of brain responses**. Panel **(A)** shows grand-average time frequency representations (TFRs) measured at the occipital-parietal [top two rows, (P1+P3+P5+POz+PO3)/5] and right-frontal [bottom two rows, (Fz+F2+FCz+FC2)/4] regions of the CO, SI, and RI trial-elicited modulations of event-related spectral perturbation (ERSP) magnitude (ER%) in pretest and posttest. *X*-axis, time (s); *Y*-axis, frequency (Hz). The color scale represents the average increase or decrease of oscillation magnitude (ER%), relative to a pre-stimulus reference interval from −0.7 to −0.1 s. Panel **(B)** shows the statistical results of two-way repeated-measures ANOVA. *X*-axis, time (s); *Y*-axis, frequency (Hz). The color scale corresponds to the *F* value where the factor practice (pretest and posttest) interacts with the factor congruency (CO, SI) or (SI, RI). The right column, two-way repeated-measures ANOVA to assess the effects of congruency (CO, SI) or (SI, RI) and practice (pretest and posttest) on the grand-average TFRs of ERSP magnitude (ER%) and to define significant S-ROIs. The factor practice (pretest and posttest) significantly interacted with the factor congruency (SI, RI) (the second one) but not (CO, SI) (the first one) in the occipital-parietal region (P1, P3, P5, POz, and PO3). The factor practice (pretest and posttest) significantly interacted with the factor congruency (CO, SI) (the third one) but not (SI, RI) (the last one) in the right-frontal region (Fz, F2, FCz, and FC2). These regions were defined as the S-ROIs (marked in white shapes). The left column, two-way repeated-measures ANOVA to assess the effects of the two experimental factors on the grand-average TFRs of ERSP magnitude (ER%) and to define significant TF-ROIs within the defined S-ROIs. Note that the time-frequency points with the significance level of *p* < 0.050 (*F*_(1,30)_ > 5, false discovery rate (FDR)-corrected) for the interaction between congruency (CO, SI) or (SI, RI) and practice (pretest and posttest) were outlined in white rectangles. The alpha-band TF-ROIs (10–13 Hz, 0.49–0.9 s; 7–10 Hz, 0.45–0.85 s) were defined.

**Figure 3:**
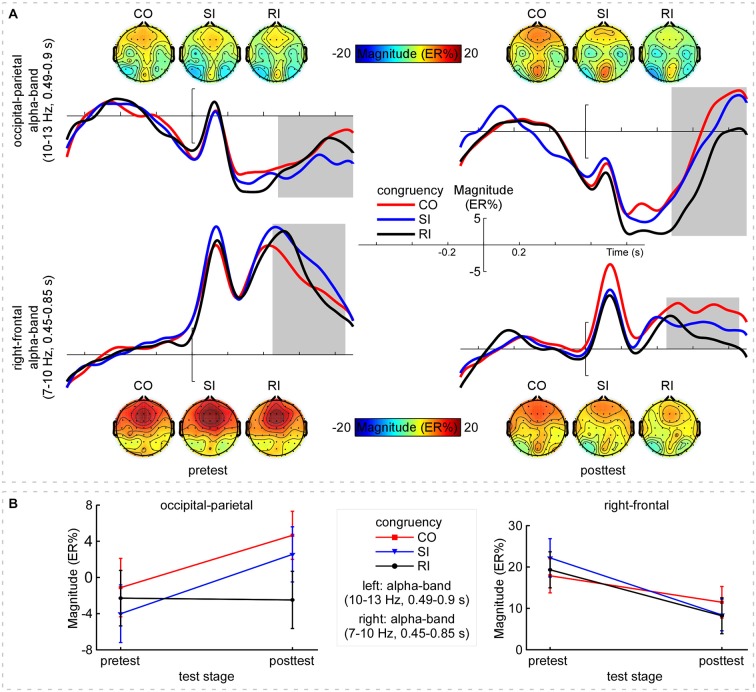
**Alpha-band magnitude**. Panel **(A)** shows the time course and scalp topographies of the alpha-band magnitude (ER%) in the pretest and posttest. *X*-axis, time (s); *Y*-axis, magnitude (ER%). The vertical bars indicate the onsets of the stimuli. **Top:** The CO, SI, and RI trial-induced ERSP magnitude in alpha band (10–13 Hz, 0.49–0.9 s, outlined in gray rectangles) distributed at the left occipital-parietal region in the pretest; while, the induced magnitude significantly decreased with practice. **Bottom:** Across practice, the CO, SI, and RI trial types induced clear ERS in alpha band (7–10 Hz, 0.45–0.85 s, outlined in gray rectangles) at the right-frontal region, whereas the ERS was markedly decreased with practice. Panel **(B)** shows mean ERSP magnitude for the CO, SI, and RI trial types in the pretest and posttest. **Left:** Alpha-band (10–13 Hz, 0.49–0.9 s) magnitude measured at the occipital-parietal region, which was enhanced for both the CO and the SI trial types but was constant for the RI trial type with practice. **Right:** Alpha-band (7–10 Hz, 0.45–0.85 s) magnitude measured at the right-frontal region. Note that the decline in alpha-band magnitude was clearly observed for all trial types with practice. Error bars indicate ±1 standard errors of the mean (SEMs). NB. “CO”, “SI”, and “RI” are the congruent, stimulus incongruent, and response incongruent trial types, respectively.

The grand-average TFRs for the CO, SI, and RI trial types in pretest and posttest were measured at the occipital-parietal (P1+P3+P5+POz+PO3)/5 and right-frontal (Fz+F2+FCz+FC2)/4 regions (Figure 2A; Table 1). Since alpha-band magnitude was widely found to be involved in the congruency tasks, we examined how alpha-band magnitude was affected by practice and trial type. The S-ROIs where the factor practice (pretest and posttest) significantly interacted with: (1) the RI-SI contrast in the occipital-parietal region; and (2) the SI-CO contrast in the right-frontal region for the ERSP magnitude were defined (marked in white shapes in Figure 2B, the right column, *F*_(1,30)_ > 5, *p* < 0.050, FDR-corrected). However, the factor practice did not significantly interact with: (1) the factor SI-CO contrast in the occipital-parietal region within 10–13 Hz, 0.49–0.9 s TF-ROI; or (2) the RI-SI contrast in the right-frontal region within 7–10 Hz, 0.45–0.85 s TF-ROI. Similarly to the RT analyses, the obtained ERSP magnitude in the alpha-band was compared with the two-way repeated-measures ANOVA. The statistical results were illustrated in Table 4. Notably, the repeating of the statistical analysis was aimed to intuitively assess the relationship of magnitude among experimental conditions, because their relationship was not easy to be detected from time-frequency representations. To intuitively illustrate the spatiotemporal features of modulations of alpha-band magnitude (ER%) for the CO, SI, and RI trial types in pre- and post-test stages, the time courses of the alpha-band magnitude (ER%) in the S-ROIs and the scalp topographies of the alpha-band magnitude (ER%) in the TF-ROIs were shown in Figure 3A. Figure 3B showed mean ERSP magnitude measured in the alpha-band [10–13 Hz, 0.49–0.9 s, occipital-parietal, left; 7–10 Hz, 0.45–0.85 s, right-frontal, right], which was a function of congruency (CO, SI, and RI) and practice (pre- and post-test).

**Table 4:** **Test statistics for the alpha-band magnitude**.

	Occipital-parietal (10–13 Hz, 0.49–0.9 s)			Right-frontal (7–10 Hz, 0.45–0.85 s)		
The main effect of congruency (CO, SI, and RI)
	*F*_(2,60)_	*p*	η^2^	*F*_(2,60)_	*p*	η^2^
	3.52	0.036	0.105	0.448	0.641	0.015
The main effect of practice (pretest and posttest)
	*F*_(1,30)_	*p*	η^2^	*F*_(1,30)_	*p*	η^2^
	2.61	0.117	0.080	33.25	***	0.526
The interaction between the two factors
	*F*_(2,60)_	*p*	η^2^	*F*_(2,60)_	*p*	η^2^
	4.63	0.014	0.134	2.84	0.066	0.087
*Post hoc* tests
	posttest: CO > RI, *p* = 0.002; SI > RI, *p* = 0.031;			CO: pretest > posttest, *p* = 0.028;		
	CO: posttest > pretest, *p* = 0.043;			SI and RI: pretest > posttest ***;		
	SI: posttest > pretest, *p* = 0.032;					

Note: ***p < .001, N = 31. “CO”, “SI”, and “RI” are the congruent, stimulus incongruent, and response incongruent trial types, respectively.

For the upper alpha-band (10–13 Hz, 0.49–0.9 s) in the occipital-parietal region, we conducted the two-way repeated-measures ANOVA with SI-CO contrast and practice as factors. The results only indicated a significant interaction between the two factors, *F*_(1,30)_ = 6.82, *p* = 0.014, η^2^ = 0.185, which was embodied in significantly stronger magnitude: (1) for SI relative to RI in posttest; and (2) for SI in posttest relative to pretest, all *p* < 0.050. Altogether, the difference in alpha-band magnitude (RI-SI) was significantly more negative in posttest compared to pretest, *t*_(30)_ = −2.61, *p* = 0.014; however, the SC (SI-CO) kept constant, *t*_(30)_ = 0.31, *p* = 0.759 (paired-samples *t* test, two-tailed). For the lower alpha-band (7–10 Hz, 0.45–0.85 s) in the right-frontal region, we conducted the two-way repeated-measures ANOVA with SI-CO contrast and practice as factors. The results indicated a significant main effect of practice, *F*_(1,30)_ = 19.80, *p* < 0.001, η^2^ = 0.368, and a significant interaction between the two factors, *F*_(1,30)_ = 6.02, *p* = 0.020, η^2^ = 0.167. Such an interaction was embodied in significantly stronger magnitude for the CO and SI in pretest relative to posttest, *p* < 0.028. Altogether, the difference in alpha-band magnitude (SI-CO) was significantly more negative in posttest relative to pretest, *t*_(30)_ = −2.45, *p* = 0.020; however, the RC (RI-SI) kept constant, *t*_(30)_ = 0.743, *p* = 0.463 (paired-samples *t* test, two-tailed).

**Figure 4:**
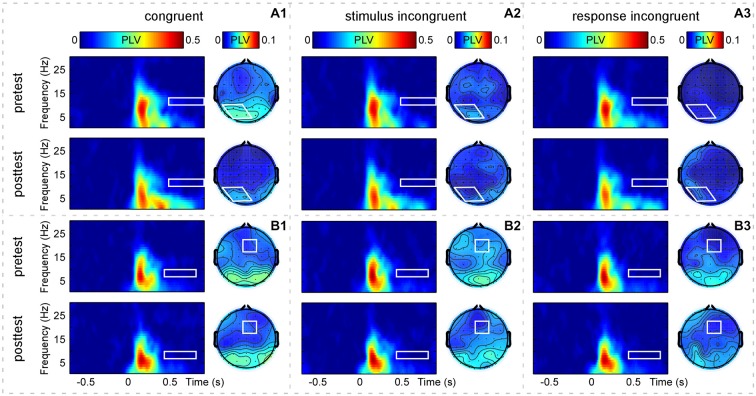
**Group-level average phase-locking value (PLV) of brain responses**. Panels **(A1–A3)** display time-frequency distributions of PLV recorded at the occipital-parietal region [(P1+P3+P5+POz+PO3)/5, outlined in white rectangles] and scalp topographies measured at the corresponding TF-ROI (10–13 Hz, 0.49–0.9 s, outlined in white shapes) for the CO, SI, and RI trial types in the pretest and posttest. Panels **(B1–B3)** display time-frequency distributions of PLV recorded at the right-frontal region [(Fz+F2+FCz+FC2)/4, outlined in white rectangles] and scalp topographies measured at the corresponding TF-ROI (7–10 Hz, 0.45–0.85 s, outlined in white rectangles) for the CO, SI, and RI trial types in the pretest and posttest. It is notable that the alpha-band oscillations are non-phase-locked to the presentation of the stimuli. *X*-axis, time (s); *Y*-axis, frequency (Hz). The color scale represents the average increase of PLV to the onset of the stimulus, relative to a pre-stimulus reference interval from −0.7 to −0.1 s.

In addition, Figure 4 showed the grand-average PLV of brain responses measured at the defined S-ROIs (occipital-parietal (P1+P3+P5+POz+PO3)/5 and right-frontal (Fz+F2+FCz+FC2)/4 regions) and scalp topographies measured within the corresponding TF-ROIs (10–13 Hz, 0.49–0.9 s; 7–10 Hz, 0.45–0.85 s; marked using white rectangles) for the three trial types in pretest and posttest. The two-way repeated-measures ANOVA with congruency and practice as factors was used to compare the PLV in each defined TF-ROI measured at the corresponding S-ROI. For the PLV in the upper alpha-band TF-ROI (10–13 Hz, 0.49–0.9 s), the two-way repeated-measures ANOVA only revealed a significant main effect of congruency, *F*_(2,60)_ = 6.51, *p* = 0.003, η^2^ = 0.178. For the PLV in the lower alpha-band TF-ROI (7–10 Hz, 0.45–0.85 s), the two-way repeated-measures ANOVA did not indicate any significant effect (*p* > 0.381). As displayed by Figures 2A and 4, the brain responses in the alpha-band TF-ROIs (10–13 Hz, 0.49–0.9 s; 7–10 Hz, 0.45–0.85 s) were non-phase-locked to the stimulus onset for all trial types across practice, hence not detected by the conventional across-trial averaging in the time domain.

## Discussion

The main findings of the present study are summarized as follows. The behavioral results showed that practice enlarged the RC, but did not affect the size of the SC. The ERP results showed that practice did not change the SI-CO contrast, but enlarged the RI-SI contrast. Similarly, the time-frequency analyses showed that the power (ER%) of the upper alpha band (10–13 Hz, 0.49–0.9 s) in the occipital-parietal region was not affected by practice on the SI-CO contrast, but enlarged on the RI-SI contrast with practice. However, the power of the lower alpha band (7–10 Hz, 0.45–0.85 s) in the right-frontal region was decreased with practice in the three conditions, and practice significantly decreased the SI-CO contrast, but did not affect the RI-SI contrast. The present behavioral result patterns replicate one previous study (Chen et al., 2013b), suggesting that the critical findings (practice enlarges RC in the flanker task, but does not affect SC) are reliable. Importantly, the results of ERPs and neural oscillations in the present study provide direct data to account for the behavioral performance.

Although the results of P3 amplitudes seem corresponding to those of RT, a careful inspection told us that practice did not change the P3 amplitudes for the CO and SI trial types, but enlarged those for the RI trial type (Figure 1B). Previous studies have shown that P3 generation reflected a process of response inhibition (Enriquez-Geppert et al., 2010; Frühholz et al., 2011) or was involved in monitoring whether the decision to classify some stimulus was appropriately transformed into action (Verleger et al., 2005). Therefore, the result that the practice enlarged the P3 amplitudes only in the RI trial type suggested that the P3 component might reflect a control for the RC, but not for the SC. Further, the increasing RC may lead the brain to recruit more attentional resources to improve cognitive control (Polich, 2007; Clayson and Larson, 2011). Given that the P3 amplitudes of the CO and SI trial types were hardly affected by practice, the P3 component should not reflect general practice effects on the basic processing, such as stimulus processing and response execution. Thus, practice-related effects on the stimulus processing and response execution are likely to be reflected on the neural oscillations.

The neural oscillation results showed that the practice-related modulation of the upper alpha-band activity in the occipital-parietal region reflected the modulation of the RC rather than SC, such a pattern was superficially consistent with the findings of P3 amplitude. Additionally, the practice-related modulations for the three trial types were virtually opposite, because practice almost synchronously enlarged the upper alpha magnitude for the CO and SI trial types, but did not change it for the RI trial types (Figure 3B, left). It is notable that the upper alpha activity has been correlated with search and retrieval processes in long-term memory (Klimesch et al., 1997; Klimesch, 1999). In the present study, stimuli were arbitrarily assigned to responses and, therefore, with practice, the associations between stimuli and responses should be weak in the pretest but be strong in the posttest. Meanwhile, as practice is a learning process, by which stimulus-response (S-R) associations could be strengthened gradually, and the learned S-R associations may be stored in the long-term memory, the upper alpha-band magnitude modulation in the occipital-parietal region may index the enhanced S-R association with practice. Especially, for the CO trial type where there was no any conflict, the decreased upper alpha ERD might exclusively reflect the enhanced S-R association. Taken together, the upper alpha-band magnitude modulation may reflect the enhanced response execution, or the association between semantic code (on the intermedial layer) and response output (Cohen et al., 1990; Herd et al., 2006).

By contrast, the practice-related modulation of the lower alpha-band activity in the right-frontal region reflected the modulation of the SC rather than RC, which was validated by: (1) the lower alpha-band ERS magnitude was decreased for the three trial types with practice (Figure 3B, right); (2) the decreased magnitude was larger for the SI relative to CO trial type; and (3) the lower alpha-band magnitude was comparable for the SI and RI trial types. Together, the lower alpha may be sensitive to the conflict and non-conflict, while, it may not differentiate the difference between the SC and the RC.

It is known that the lower alpha-band ERD is critically related to the attentional demands such as alertness (Klimesch et al., 2007). Moreover, the modulation of the lower alpha-band magnitude was most pronounced in the right-frontal region, which suggests an endogenous control processing (Wu et al., 2015). The evidences from functional magnetic resonance imaging (fMRI) studies have showed that the right anterior insula is an important part of the salience network (Seeley et al., 2007), by which the brain can monitor the salience of the input information, such as conflict and error signals (Bressler and Menon, 2010). Therefore, we propose that with practice, the lower alpha-band magnitude modulation in the right-frontal region may reflect an enhancement in alertness when conflict occurs. In this case, the brain may have adapted to the conflict information and, therefore, decreases the conflict-related activity. Furthermore, the relative small lower alpha modulation for the CO trial type (here no conflict occurrence) also supports that this modulation is related to the alertness to conflict occurrence. Together, practice may decrease the sensitiveness of the brain for conflict occurrence, regardless of the type of the conflict (SC or RC).

## Conclusion

In conclusion, the present study shows that the practice-related effects on both RT and P3 amplitudes are similar: with practice, the RT and P3 amplitudes for the RI trial type are increased, while, those are not changed for the CO and SI trial types. The neural oscillation results show that the practice-related modulation of the upper alpha-band power in the occipital-parietal region reflects an enhanced S-R association, which accounts for the enhanced RC in behavioral performances. The lower alpha-band magnitude modulation reflects the effect of practice on the SC but not on the RC, suggesting a decreased alertness or sensitiveness to the occurrence of conflict with practice. Therefore, the current study demonstrates the neural mechanisms of the occurrence of both SC and RC in the flanker task by using a practice paradigm.

## Conflict of Interest Statement

The authors declare that the research was conducted in the absence of any commercial or financial relationships that could be construed as a potential conflict of interest.
